# A differentiated approach to referrals from general practice to support early cancer diagnosis – the Danish three-legged strategy

**DOI:** 10.1038/bjc.2015.44

**Published:** 2015-03-03

**Authors:** P Vedsted, F Olesen

**Affiliations:** 1 Research Unit for General Practice, The Research Centre for Cancer Diagnosis in Primary Care (CaP), Institute of Public Health, Aarhus University, Bartholins Alle 2, 8000 Aarhus C, Denmark

**Keywords:** Denmark, diagnosis, health services organisation, access, investigation

## Abstract

When aiming to provide more expedited cancer diagnosis and treatment of cancer at an earlier stage, it is important to take into account the symptom epidemiology throughout the pathway, from first bodily sensation until the start of cancer treatment. This has implications for how primary-care providers interpret the presentation and decisions around patient management and investigation. Symptom epidemiology has consequences for how the health-care system might best be organised. This paper argues for and describes the organisation of the Danish three-legged strategy in diagnosing cancer, which includes urgent referral pathways for symptoms suspicious of a specific cancer, urgent referral to diagnostic centres when we need quick and profound evaluation of patients with nonspecific, serious symptoms and finally easy and fast access to ‘No-Yes-Clinics' for cancer investigations for those patients with common symptoms in whom the diagnosis of cancer should not be missed. The organisation of the health-care system must reflect the reality of symptoms presented in primary care. The organisational change is evaluated and monitored with a comprehensive research agenda, data infrastructure and education.

In recent years, many health-care systems have implemented specific strategies to ensure timely cancer diagnosis (Department of Health, 2000; Prades *et al*, 2011). This has been motivated by poor cancer control, public discontent with long waiting times and an organisational and economic attempt for efficiency in standardised diagnostic pathways (Richards, 2009). Reports have shown that cancer survival in the United Kingdom and Denmark is lower than that in other countries (Storm *et al*, 2010; Coleman *et al*, 2011). Danish cancer patients are treated at later stages (Maringe *et al*, 2012; Walters *et al*, 2013a, 2013b), suggesting delays in presentation, diagnosis and treatment. This is supported by the evidence that waiting times can be long in the Danish cancer care system, which may lead to higher mortality (Tørring *et al*, 2011, 2012, 2013; Elit *et al*, 2014; Redaniel *et al*, 2014; Neal *et al*, 2015) and stage progression (Jensen *et al*, 2007; Wang *et al*, 2012).

In 2008, after several years of investment in cancer treatment and two cancer plans, Denmark introduced urgent referral for suspected cancer (Olesen *et al*, 2009). Politically, cancer was proclaimed an acute disease for which diagnosis and treatment should be without waiting time (Probst *et al*, 2012). Such urgent referral pathways are in place in a number of health-care systems and are being developed in others. Based on the defined alarm symptoms, the GP can suspect cancer and refer urgently to a specific pathway, and the speed and logistics of the diagnostic pathway and the standardisation of treatment within the hospital setting can be improved (Toustrup *et al*, 2011; Vallverdú-Cartié *et al*, 2011; Valentín-López *et al*, 2012; Dyrop *et al*, 2013; Larsen *et al*, 2013).

In the process that followed the introduction of the urgent referral pathway in Denmark, it became obvious that this pathway was inadequate to ensure timely diagnosis of all cancer patients, and a strategy based on three cornerstones was developed.

This paper argues for and describes the three-legged strategy for cancer diagnosis in Denmark, where the purpose is to accommodate the fact that patients with an early-stage cancer present very differently in general practice and that a single focus on alarm symptoms or red flags might not be sufficient.

## Reasons for longer diagnostic pathways

If cancer patients are asked, after treatment completion, what they consider the most important part of the pathway, they report aspects of expedited referral, diagnosis and treatment and short waiting time for all investigations, including the very first ones often prescribed by the GP (Booji *et al*, 2013). This is also indicated by the finding that Danish cancer patients' confidence in their GPs decreases with increasing time to diagnosis (Larsen *et al*, 2011).

A study among Danish GPs in 2010, after the introduction of urgent referral for suspected cancer, asked about their cancer patients, diagnostic pathways, and showed that in about one-third of cases, the GPs reported a quality deviation which was strongly associated with longer diagnostic intervals (Jensen *et al*, 2014).

There is also evidence that the organisation of the health-care system may have adverse effects. In an ecological study in which we compared a number of European countries' primary care and their 1-year cancer survival rates, we saw that countries with a strong gatekeeper role also had the lowest cancer survival rates (Vedsted and Olesen, 2011). This could suggest that in some countries where GPs were good gatekeepers, the GPs had become too reluctant to refer early to diagnostic investigations. Further, that access to diagnostic services in the initial phase was slow or rationed, resulting in patients not obtaining timely cancer investigations.

## Symptom epidemiology and the ‘diagnostic funnel'

**Figure 1:**
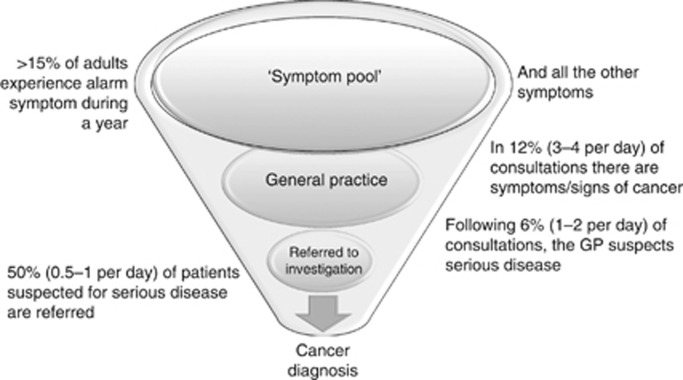
**The diagnostic funnel showing the symptom epidemiology from public to cancer diagnosis.** General practice is placed between the public ‘symptom pool' and the decision to investigate for cancer.

Another aspect is that health-care systems should be organised to support the diagnostic needs. In cancer diagnosis, the processes related to symptom epidemiology must be recalled (Figure 1; Elliott *et al*, 2011). In line with this, studies have demonstrated the 'symptom iceberg', showing that >15% of adults will have experienced at least one cancer alarm symptom during the last year (Svendsen *et al*, 2010). Only a small proportion of these will seek help, for example 10% of those with rectal bleeding consult their GP (McAvoy, 2007).

Despite relevant screening activities, ∼85–90% of all cancers are diagnosed on the basis of symptomatic presentation (Hansen *et al*, 2011; Vedsted and Olesen, 2009; Emery *et al*, 2014). In health-care systems in which GPs form a specialised first line, data show that 75–85% of all cancer patients start in general practice by presenting signs or symptoms (Allgar and Neal, 2005).

It has been shown that the GP indicates potential alarm symptoms in up to 12% (Ingebrigsten *et al*, 2013) of all consultations and suspect a serious disease in need of further elucidation in 6% of consultations (Hjertholm *et al*, 2014) (Figure 1). A study showed that 10% of these patients had a new serious diagnosis within 2 months. This means that when the GP suspects serious illness there are reasons to support the GP in having access to relevant investigations (Nylenna, 1986).

## The symptom continuum

**Figure 2:**
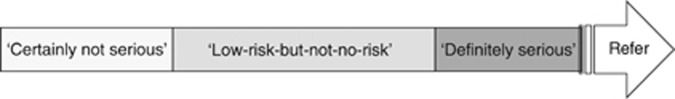
**The symptom continuum in general practice.** A symptom can present clinically in many ways.

Another important aspect is that, once a symptom is presented in general practice, the severity of a symptom could be thought of as forming a continuum. An example of this continuum could be abdominal pain. In the clinical context, abdominal pain should be understood in terms of the continuum with increasing clinical significance, thus becoming more and more indicative of a serious disease (cancer). In the clinical cancer diagnosis, a symptom does not present as ‘there' or ‘not there'. Symptoms appear from ‘certainly not serious' to ‘definitely serious' (Figure 2).

The first section (left) of the symptom continuum is that in which the symptom presents as harmless. The second section is where the symptom is most probably not a sign of cancer, although cancer cannot be excluded. This is the so-called ‘low-risk-but-not-no-risk' symptom (Hamilton, 2010). The third section is where the symptom definitely indicates risk of a serious disease and an urgent referral is relevant. Fortunately, in general practice it is rarely cancer, even if it is an alarm symptom (Hamilton, 2009a). However, it is still the GP's duty to identify the cancer over the whole symptom continuum. Progression along the symptom continuum might, for example, be the reason that we can observe that patients who are later diagnosed with cancer tend to see their GP several months before diagnosis (Christensen *et al*, 2012; Ahrensberg *et al*, 2013). Here the GPs may have used time as a diagnostic test (Almond and Summerton, 2009). However, waiting until the symptom becomes definitely serious might also imply a stage progression in some cancers.

## The ‘obvious', ‘difficult' and ‘common' cancer presentations

A study in Danish general practice has revealed that, if GPs are allowed simply to categorise the first presentation of cancer, 50% are categorised as alarm symptoms, 20% are serious, but cancer nonspecific symptoms and 30% are categorised as normal vague symptoms (Jensen *et al*, 2014). This has also been supported by the finding that 50% of cancer patients in United Kingdom general practice did not have a National Institute for Health and Care Excellence guideline symptom suspicious for cancer registered in the patient record (Neal *et al*, 2014). Thus, some cancer patients do present in general practice, but not with symptoms indicative of cancer.

## The three-legged diagnostic strategy

A wish for expedited cancer diagnosis and cancer diagnosis at an earlier stage necessitates that we broaden the focus on alarm symptoms to include the full symptom continuum. A system that focusses on cancer diagnosis based on alarm symptoms alone might also be a health-care system that favours ‘the sick-quick' and lets down the majority with vague symptoms (Hamilton, 2009a). A Danish study illustrated this by showing that, if the GP regards the symptom as vague, 50% of cancer patients will wait at least one month more and 25% at least 2.5 months longer until diagnosis, compared with those with alarm symptoms (Jensen *et al*, 2014). Thus, urgent referral for the obvious alarm symptoms must be accompanied by two more referral routes; the urgent referral for nonspecific, serious symptoms and the no-yes-clinics (NYC).

**Figure 3:**
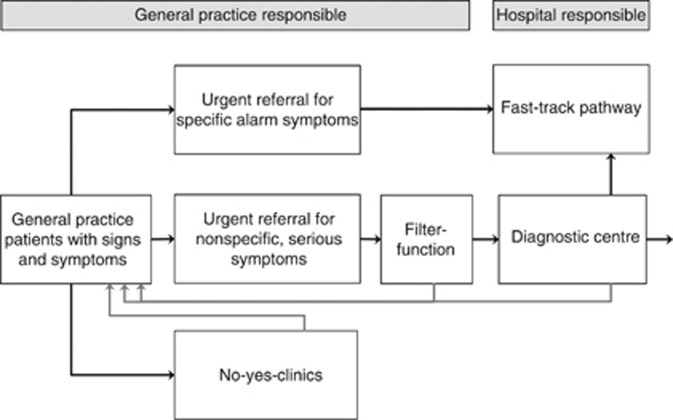
The structure of the Danish three-legged diagnostic strategy.

This led to the development of the three-legged cancer diagnosis strategy in Denmark (Figure 3). It acknowledges that we need diagnostic routes for what the GPs recognise as alarm symptoms (the obvious cancer suspicion), the nonspecific symptoms (the difficult diagnosis) and the vague symptoms (the common symptom).

## The urgent referral pathway

From UK-based practice research, it is known that the risk of having cancer given a single alarm symptom is often in the range of 3–8% (Jones *et al*, 2007; Hamilton, 2009b; Shapley *et al*, 2010). Meechan and colleagues (2012) showed that, among those referred to the urgent referral pathway, the risk of cancer was 11%. Thus, the urgent referral strategy seems to be effective. However, what is also shown – and forms the platform for introducing further diagnostic possibilities – is that on average only 40–45% (with differences between cancer types) of all cancer patients are primarily referred to specific pathways (Meechan *et al*, 2012; Jensen *et al*, 2014). This means that the largest group of cancer patients is not offered this faster and perhaps most appropriate route to diagnosis (Elliss-Brookes *et al*, 2012; Guldbrandt *et al*, 2015).

## Urgent referral for unspecific, serious symptoms and the diagnostic centres

The urgent referral for unspecific, serious symptoms was implemented nationally by the National Board of Health and Danish Regions in 2012. When a GP has a patient that is clearly sick, and where cancer is one of several diagnostic possibilities, they can be referred. The pathway consists of a two-step approach with a filter function performed by the GP and, if still relevant, a referral to a diagnostic centre. The filter function is a standard battery of diagnostic investigations consisting of blood and urine tests and diagnostic imaging. The results of the investigations are sent electronically to the GP within four working days. The GP subsequently decides further diagnostic steps within eight working days. If there is no explanation for the symptoms the GP can refer to the diagnostic centre and no longer has the diagnostic responsibility for the patient.

A diagnostic centre is a medical unit with comprehensive facilities for medical investigation, including easy access to expertise in a wide range of relevant specialities. Patients are appointed a responsible doctor for the outpatient trajectory.

Each of the five Danish regions must have at least one diagnostic centre, and ∼15 centres have now been established. The symptoms most often seen at referral are weight loss, fatigue, unspecific pain and nausea. ‘Problems with general health' and ‘GP's gut feeling' are among the most likely clinical signs for referral to a diagnostic centre. The proportion with cancer among those referred is ∼15–20%. The cancers most often seen are lung, colorectal and haematological cancers. There are ongoing publications of these specific results.

## The NYC

For the 30–40% of cancer patients with vague, ‘low-risk-but-not-no-risk', symptoms, Denmark now, by a governmental regulation, implements the ‘NYC'. These are services conducted in hospitals or specialist clinics. The GP has direct access to fast investigations as part of the classical iterative diagnostic process (Norman *et al*, 2009) where the GP can raise a possibility of cancer – the serious diagnosis that the GP does not want to fail to spot.

The principle is to keep it simple; the GP is responsible for the diagnostic actions and the patient is not admitted to the hospital, thus avoiding use of resources, for example, history taking, blood tests, patient records and other administrative or clinical resources demanding activity. Studies indicate that the strategy can be both effective and efficient. A Dutch study of direct access to colonoscopy for abdominal symptoms showed reduced time to diagnosis and more efficient use of tests (Klemann *et al*, 2011). This is supported by studies from the United Kingdom (Maruthachalam *et al*, 2005; Ahmed *et al*, 2013). A randomised Danish study giving GPs direct access to a low-dose CT scan for suspected lung cancer showed that the use of CT scans did not increase, compared with the usual ‘double gatekeeping' by the lung specialist (Guldbrandt *et al*, 2013).

There are ongoing studies on the specific ways of organising this, which patients to refer and what investigations should be provided.

## Conclusion and perspectives

When aiming to provide more expedited cancer diagnosis and treatment of cancer at an earlier stage, it is important to take into account the symptom epidemiology throughout the pathway, from the first bodily sensation until the start of cancer treatment. This has implications for how primary care providers interpret the presentation and decisions around patient management and investigation. Symptom epidemiology has consequences for how the health-care system might best be organised.

This paper provides several reasons to support and test a three-legged strategy, in particular for diagnosing cancer in earlier stages. The GPs need diagnostic routes that, first, take into account the fact that symptoms present on a continuum from ‘certainly not serious' to ‘definitely serious' and, second, that cancers present with symptoms that sometimes obviously indicate cancer but for the majority are nonspecific and serious or vague and common. Since our previous paper in 2009 about the Danish example (Olesen *et al*, 2009), the focus in Denmark has been to develop broad support for the GPs' different tasks in diagnosing cancer. The continuous growing evidence on cancer diagnosis is implemented into daily clinical practice and health policy to ensure structural support. Thus, it should be remembered that in a modern health-care system the best pathways from symptom to cancer treatment are established only if there is a culture of responsibility – making these changes requires political, administrative and clinical leadership.

To improve and optimise this differentiated approach we call for a large research agenda and precise evidence-based implementation strategies. The effectiveness and efficiency of the diagnostic centres and the NYC need further research and monitoring, and the Danish government and regions are engaged in this work. Intervention studies are needed to test whether there is an effect on stage distribution and survival, quality of life, health economics and patient evaluation. There is a need for more clinical research, including research into primary care and specialised diagnostic investigations. Finally, we must also ensure practical implementation by education (Guldbrandt *et al*, 2014) and facilities for primary diagnosis (Toftegaard *et al*, 2014).

## Notes

FO is the chairman of the Danish Cancer Society (2015). PV declares no conflict of interest.

## References

[bib1] Ahmed J Mehmood S Khan SA Rao MM 2013 Direct access colonoscopy in primary care: is it a safe and practical approach Scott Med J 58 168 172 23960056 10.1177/0036933013496963

[bib2] Ahrensberg JM Fenger-Grøn M Vedsted P 2013 Use of primary care during the year before childhood cancer diagnosis: a nationwide population-based matched comparative study PLoS One 8 (3 e59098 23554980 10.1371/journal.pone.0059098 PMC3595276

[bib3] Allgar VL Neal RD 2005 Delays in the diagnosis of six cancers: analysis of data from the National Survey of NHS Patients Br J Cancer 92 1959 1970 15870714 10.1038/sj.bjc.6602587 PMC2361797

[bib4] Almond SC Summerton N 2009 Test of time BMJ 338 b1878 19528115 10.1136/bmj.b1878

[bib5] Booij JC Zegers M Evers PM Hendriks M Delnoij DM Rademakers JJ 2013 Improving cancer patient care: development of a generic cancer consumer quality index questionnaire for cancer patients BMC Cancer 13 203 23617741 10.1186/1471-2407-13-203 PMC3648393

[bib6] Christensen KG Fenger-Grøn M Flarup KR Vedsted P 2012 Use of general practice, diagnostic investigations and hospital services before and after cancer diagnosis—a population-based nationwide registry study of 127,000 incident adult cancer patients BMC Health Serv Res 12 224 22838741 10.1186/1472-6963-12-224 PMC3507912

[bib7] Coleman MP Forman D Bryant H Butler J Rachet B Maringe C Nur U Tracey E Coory M Hatcher J McGahan CE Turner D Marrett L Gjerstorff ML Johannesen TB Adolfsson J Lambe M Lawrence G Meechan D Morris EJ Middleton R Steward J Richards MA ICBP Module 1 Working Group 2011 Cancer survival in Australia, Canada, Denmark, Norway, Sweden, and the UK, 1995-2007 (the International Cancer Benchmarking Partnership): an analysis of population-based cancer registry data Lancet 377 127 138 21183212 10.1016/S0140-6736(10)62231-3 PMC3018568

[bib8] Department of Health 2000 The NHS Cancer Plan A Plan for Investment, a plan for reform Department of Health: London, UK

[bib9] Dyrop HB Safwat A Vedsted P Maretty-Nielsen K Hansen BH Jørgensen PH Baad-Hansen T Bünger C Keller J 2013 Cancer Patient Pathways shortens waiting times and accelerates the diagnostic process of suspected sarcoma patients in Denmark Health Policy 113 110 117 24138955 10.1016/j.healthpol.2013.09.012

[bib10] Elit LM O'Leary EM Pond GR Seow HY 2014 Impact of wait times on survival for women with uterine cancer J Clin Oncol 32 27 33 24276779 10.1200/JCO.2013.51.3671

[bib11] Elliott AM McAteer A Hannaford PC 2011 Revisiting the symptom iceberg in today's primary care: results from a UK population survey BMC Fam Pract 12 16 21473756 10.1186/1471-2296-12-16 PMC3083353

[bib12] Elliss-Brookes L McPhail S Ives A Greenslade M Shelton J Hiom S Richards M 2012 Routes to diagnosis for cancer—determining the patient journey using multiple routine data sets Br J Cancer 107 1220 1226 22996611 10.1038/bjc.2012.408 PMC3494426

[bib13] Emery JD Shaw K Williams B Mazza D Fallon-Ferguson J Varlow M Trevena LJ 2014 The role of primary care in early detection and follow-up of cancer Nat Rev Clin Oncol 11 38 48 24247164 10.1038/nrclinonc.2013.212

[bib14] Guldbrandt LM Fenger-Grøn M Folkersen BH Rasmussen TR Vedsted P 2013 Reduced specialist time with direct computed tomography for suspected lung cancer in primary care Dan Med J 60 A4738 24355447

[bib15] Guldbrandt L Fenger-Grøn M Rasmussen T Jensen H 2015 The role of general practice in routes to diagnosis of lung cancer in Denmark: a population-based study of general practice involvement, diagnostic activity and diagnostic intervals BMC Health Serv Res 2215(1):21 25608462 10.1186/s12913-014-0656-4 PMC4307896

[bib16] Guldbrandt LM Rasmussen TR Rasmussen F Vedsted P 2014 Implementing direct access to low-dose computed tomography in general practice-method, adaption and outcome PLoS One 9 e112162 25383780 10.1371/journal.pone.0112162 PMC4226510

[bib17] Hamilton W 2009 Five misconceptions in cancer diagnosis Br J Gen Pract 59 441 445 19520027 10.3399/bjgp09X420860 PMC2688046

[bib18] Hamilton W 2009 The CAPER studies: five case-control studies aimed at identifying and quantifying the risk of cancer in symptomatic primary care patients Br J Cancer 101 S80 S86 19956169 10.1038/sj.bjc.6605396 PMC2790706

[bib19] Hamilton W 2010 Cancer diagnosis in primary care Br J Gen Pract 60 121 128 20132704 10.3399/bjgp10X483175 PMC2814263

[bib20] Hansen R Vedsted P Sokolowski I Søndergaard J Olesen F 2011 Time intervals from first symptom to treatment of cancer: a cohort study of 2,212 newly diagnosed cancer patients BMC Health Serv Res 11 284 22027084 10.1186/1472-6963-11-284 PMC3217887

[bib21] Hjertholm P Moth G Ingeman ML Vedsted P 2014 Predictive values of GPs' suspicion of serious disease: a population-based follow-up study Br J Gen Pract 64 346 353 10.3399/bjgp14X680125 PMC4032017 24868072

[bib22] Ingebrigtsen SG Scheel BI Hart B Thorsen T Holtedahl K 2013 Frequency of 'warning signs of cancer' in Norwegian general practice, with prospective recording of subsequent cancer Fam Pract 30 153 160 23097250 10.1093/fampra/cms065

[bib23] Jensen AR Nellemann HM Overgaard J 2007 Tumor progression in waiting time for radiotherapy in head and neck cancer Radiother Oncol 84 5 10 17493700 10.1016/j.radonc.2007.04.001

[bib24] Jensen H Nissen A Vedsted P 2014 Quality deviations in cancer diagnosis: prevalence and time to diagnosis in general practice Br J Gen Pract 64 e92 e98 24567622 10.3399/bjgp14X677149 PMC3905405

[bib25] Jensen H Tørring ML Olesen F Overgaard J Vedsted P 2014 Cancer suspicion in general practice, urgent referral and time to diagnosis: a population-based GP survey and registry study BMC Cancer 14 636 25175155 10.1186/1471-2407-14-636 PMC4164756

[bib26] Jones R Latinovic R Charlton J Gulliford MC 2007 Alarm symptoms in early diagnosis of cancer in primary care: cohort study using General Practice Research Database BMJ 334 1040 17493982 10.1136/bmj.39171.637106.AE PMC1871798

[bib27] Klemann VM Wolters FL Konsten JL 2011 Benefits of a well-structured diagnostic process in colon cancer Dig Surg 28 15 21 21293127 10.1159/000321894

[bib28] Larsen MB Hansen RP Hansen DG Olesen F Vedsted P 2013 Secondary care intervals before and after the introduction of urgent referral guidelines for suspected cancer in Denmark: a comparative before-after study BMC Health Serv Res 13 348 24021054 10.1186/1472-6963-13-348 PMC3847112

[bib29] Larsen MB Hansen RP Olesen F Vedsted P 2011 Patients' confidence in their GP before and after being diagnosed with cancer Br J Gen Pract. 61 e215 e222 21619745 10.3399/bjgp11X572409 PMC3080226

[bib30] Maringe C Walters S Butler J Coleman MP Hacker N Hanna L Mosgaard BJ Nordin A Rosen B Engholm G Gjerstorff ML Hatcher J Johannesen TB McGahan CE Meechan D Middleton R Tracey E Turner D Richards MA Rachet B ICBP Module 1 Working Group 2012 Stage at diagnosis and ovarian cancer survival: Evidence from the International Cancer Benchmarking Partnership Gynecol Oncol 127 75 82 22750127 10.1016/j.ygyno.2012.06.033

[bib31] Maruthachalam K Stoker E Chaudhri S Noblett S Horgan AF 2005 Evolution of the two-week rule pathway—direct access colonoscopy vs outpatient appointments: one year's experience and patient satisfaction survey Colorectal Dis 7 480 485 16108885 10.1111/j.1463-1318.2005.00868.x

[bib32] McAvoy B 2007 General practitioners and cancer control Med J Aust 187 115 117 17635097 10.5694/j.1326-5377.2007.tb01156.x

[bib33] Meechan D Gildea C Hollingworth L Richards MA Riley D Rubin G 2012 Variation in use of the 2-week referral pathway for suspected cancer: a cross-sectional analysis Br J Gen Pract 62 e590 e597 22947579 10.3399/bjgp12X654551 PMC3426597

[bib34] Neal RD Din NU Hamilton W Ukoumunne OC Carter B Stapley S Rubin G 2014 Comparison of cancer diagnostic intervals before and after implementation of NICE guidelines: analysis of data from the UK General Practice Research Database Br J Cancer 110 584 592 24366304 10.1038/bjc.2013.791 PMC3915139

[bib35] Neal RD Tharmanathan P France B Din NU Cotton S Fallon-Ferguson J Hamilton W Hendry A Hendry M Lewis R Macleod U Mitchell ED Pickett M Rai T Shaw K Stuart N Tørring ML Wilkinson C Williams B Williams N Emery J 2015 Is increased time to diagnosis and treatment in symptomatic cancer associated with poorer outcomes? Systemic Review Br J Cancer 112 S92 S107 25734382 10.1038/bjc.2015.48 PMC4385982

[bib36] Norman G Barraclough K Dolovich L Price D 2009 Iterative diagnosis BMJ 339 b3490 19773326 10.1136/bmj.b3490

[bib37] Nylenna M 1986 Diagnosing cancer in general practice: from suspicion to certainty Br Med J (Clin Res Ed) 293 314 317 10.1136/bmj.293.6542.314 PMC1340993 3089502

[bib38] Olesen F Hansen RP Vedsted P 2009 Delay in diagnosis: the experience in Denmark Br J Cancer 101 (Suppl 2 S5 S8 19956163 10.1038/sj.bjc.6605383 PMC2790711

[bib39] Prades J Espinàs JA Font R Argimon JM Borràs JM 2011 Implementing a cancer fast-track programme between primary and specialised care in Catalonia (Spain): a mixed methods study Br J Cancer 105 753 759 21829194 10.1038/bjc.2011.308 PMC3171014

[bib40] Probst HB Hussain ZB Andersen O 2012 Cancer patient pathways in Denmark as a joint effort between bureaucrats, health professionals and politicians—a national Danish project Health Policy 105 65 70 22136810 10.1016/j.healthpol.2011.11.001

[bib41] Redaniel MT Martin RM Blazeby JM Wade J Jeffreys M 2014 The association of time between diagnosis and major resection with poorer colorectal cancer survival: a retrospective cohort study BMC Cancer 14 642 25175937 10.1186/1471-2407-14-642 PMC4159515

[bib42] Richards MA 2009 The national awareness and early diagnosis initiative in England: assembling the evidence Br J Cancer 101 (Suppl 2 S1 S4 19956152 10.1038/sj.bjc.6605382 PMC2790704

[bib43] Shapley M Mansell G Jordan JL Jordan KP 2010 Positive predictive values of ⩾5% in primary care for cancer: systematic review Br J Gen Pract 60 e366 e377 20849687 10.3399/bjgp10X515412 PMC2930247

[bib44] Storm HH Kejs AM Engholm G Tryggvadóttir L Klint A Bray F Hakulinen T 2010 Trends in the overall survival of cancer patients diagnosed 1964-2003 in the Nordic countries followed up to the end of 2006: the importance of case-mix Acta Oncol 49 713 724 20491527 10.3109/0284186X.2010.484426

[bib45] Svendsen RP Støvring H Hansen BL Kragstrup J Søndergaard J Jarbøl DE 2010 Prevalence of cancer alarm symptoms: a population-based cross-sectional study Scand J Prim Health Care. 28 132 137 20698729 10.3109/02813432.2010.505412 PMC3442327

[bib46] Toftegaard B Bro F Vedsted P 2014 A geographical cluster randomised stepped wedge study of continuing medical education and cancer diagnosis in general practice Implement Sci 9 (1 159 25377520 10.1186/s13012-014-0159-z PMC4229614

[bib47] Tørring ML Frydenberg M Hamilton W Hansen RP Lautrup MD Vedsted P 2012 Diagnostic interval and mortality in colorectal cancer: U-shaped association demonstrated for three different datasets J Clin Epidemiol 65 669 678 22459430 10.1016/j.jclinepi.2011.12.006

[bib48] Tørring ML Frydenberg M Hansen RP Olesen F Hamilton W Vedsted P 2011 Time to diagnosis and mortality in colorectal cancer: a cohort study in primary care Br J Cancer 104 934 940 21364593 10.1038/bjc.2011.60 PMC3065288

[bib49] Tørring ML Frydenberg M Hansen RP Olesen F Vedsted P 2013 Evidence of increasing mortality with longer diagnostic intervals for five common cancers: a cohort study in primary care Eur J Cancer 249 2187 2198 10.1016/j.ejca.2013.01.025 23453935

[bib50] Toustrup K Lambertsen K Birke-Sørensen H Ulhøi B Sørensen L Grau C 2011 Reduction in waiting time for diagnosis and treatment of head and neck cancer—a fast track study Acta Oncol 50 636 641 21261506 10.3109/0284186X.2010.551139

[bib51] Valentín-López B Ferrándiz-Santos J Blasco-Amaro JA Morillas-Sáinz JD Ruiz-López P 2012 Assessment of a rapid referral pathway for suspected colorectal cancer in Madrid Fam Pract 29 182 188 21976660 10.1093/fampra/cmr080

[bib52] Vallverdú-Cartié H Comajuncosas-Camp J Orbeal-Sáenz RA López-Negre JL Gris Garriga PJ Jimeno-Fraile J Hermoso-Bosch J Sánchez-Pradell C Torra-Alsina S Urgellés-Bosch J Parés D 2011 Results of implementation of a fast track pathway for diagnosis of colorectal cancer Rev Esp Enferm Dig 103 402 407 21867349 10.4321/s1130-01082011000800003

[bib53] Vedsted P Olesen F 2009 Early diagnosis of cancer—the role of general practice Scand J Prim Health Care 27 193 194 19958062 10.3109/02813430903478623 PMC3413909

[bib54] Vedsted P Olesen F 2011 Are the serious problems in cancer survival partly rooted in gatekeeper principles? An ecologic study Br J Gen Pract 61 e508 e512 21801563 10.3399/bjgp11X588484 PMC3145535

[bib55] Walters S Maringe C Coleman MP Peake MD Butler J Young N Bergström S Hanna L Jakobsen E Kölbeck K Sundstrøm S Engholm G Gavin A Gjerstorff ML Hatcher J Johannesen TB Linklater KM McGahan CE Steward J Tracey E Turner D Richards MA Rachet B ICBP Module 1 Working Group 2013 Lung cancer survival and stage at diagnosis in Australia, Canada, Denmark, Norway, Sweden and the United Kingdom: a population-based study, 2004-2007 Thorax 0 1 14 10.1136/thoraxjnl-2012-202297 23399908

[bib56] Walters S Maringe C Butler J Rachet B Barrett-Lee BJ Boyages J Christiansen P Lee M Wärnberg AC Engholm G Fornander T Gjerstorff ML Johannesen TB Lawrence G McGahan CE Middleton R Steward J Tracey E Turner D Richards MA Coleman MP the ICBP Module 1 Working Group 2013 Breast cancer survival and stage at diagnosis in Australia, Canada, Denmark, Norway, Sweden and the UK, 2000-2007: a population-based study Br J Cancer 108 1195 1208 23449362 10.1038/bjc.2013.6 PMC3619080

[bib57] Wang J Wang J Mahasittiwat P Wong KK Quint LE Kong FM 2012 Natural growth and disease progression of non-small cell lung cancer evaluated with 18F-fluorodeoxyglucose PET/CT Lung Cancer 78 51 56 22841591 10.1016/j.lungcan.2012.06.010 PMC3933267

